# Myocarditis Induced by Immune Checkpoint Inhibitors: An Exploratory Review

**DOI:** 10.7759/cureus.67314

**Published:** 2024-08-20

**Authors:** Esteban Zavaleta-Monestel, Jonathan García-Montero, Adriana Anchía-Alfaro, Carolina Rojas-Chinchilla, Ricardo Quesada-Villaseñor, Sebastián Arguedas-Chacón, Monserrat Barrantes-López, Paula Molina-Sojo, Andrea Zovi, Carlos Zúñiga-Orlich

**Affiliations:** 1 Pharmacy, Hospital Clínica Bíblica, San José, CRI; 2 Pharmacy and Clinical Research, Hospital Clínica Bíblica, San José, CRI; 3 Research, Hospital Clínica Bíblica, San José, CRI; 4 Faculty of Pharmacy, Universidad de Iberoamérica, San José, CRI; 5 Hygiene, Food Safety, and Nutrition, Ministry of Health, Rome, ITA; 6 Oncology, Hospital Clínica Bíblica, San José, CRI

**Keywords:** antibodies monoclonal, immunotherapy, immune system, cancer, myocarditis, checkpoint inhibitors

## Abstract

Checkpoints are essential proteins in the immune system that regulate the intensity and duration of immune responses, preventing damage to healthy tissues during the fight against pathogens and abnormal cells. While these mechanisms are crucial in cancer defense, this disease can alter the functionality of these proteins. This is why checkpoint inhibitors have emerged as an important class of drugs to potentiate the antitumor immune response. However, it has been observed that these drugs can trigger adverse effects, among which myocarditis is one of the most prevalent. This article explores the signaling pathways associated with checkpoint inhibitors, their adverse effects, and their impact on the development of myocarditis, as well as potential therapeutic strategies.

## Introduction and background

The immune system is composed of a variety of cells and organs, playing a fundamental role in the human body by combating infections and foreign invaders. It can be divided into two main types: the innate immune system, which is inherited from parents, and the adaptive immune system, which produces antibodies to protect against bacteria, viruses, and more. These antibodies can even target complex pathologies, such as various types of cancer. Upon recognizing an antigen, the immune system signals an attack to prevent its replication and further damage [1]. Adaptive immune responses also include cell-mediated immune responses, which are crucial for the effectiveness of cancer treatments with immune checkpoint inhibitors (ICIs) [2].

Among the various components of the immune system, checkpoints play a distinct role. These proteins regulate the duration and intensity of the immune response against pathologies such as cancer, autoimmune diseases, and certain skin conditions. Checkpoints are commonly found on T cells, which are a critical part of the immune system, as well as on tumor cells [3,4].

The checkpoint proteins have a wide variety of classifications; however, the main ones and those most studied are PD-L1, found in tumor cells, and CTLA-4, most seen in T cells. Checkpoints play a crucial role in the immune system by regulating T-cell activity and managing their interactions with neighboring tumor cells, which is essential for controlling the immune response to cancer. When checkpoint proteins bind to their ligands on tumor cells or antigen-presenting cells, they send a signal that stops T-cell activity. This action is key to preventing an excessive immune response that could damage healthy tissues [3].

In essence, checkpoints act as "brakes" that control the intensity of the immune response, hence their important role. When this binding is reversed or its action is blocked, T cells can resume their antitumor activity. This means that with checkpoint inhibition, T cells can be activated and attack cancer cells in the body. When considering a pathology as complex as cancer, it has been observed over time how it often manages to evade immune system surveillance, with immune checkpoints being no exception [4].

Cancer cells often produce high levels of PD-L1 proteins, which interfere with T-cell checkpoints and prevent immune attacks, allowing the cancer cells to replicate and spread [5]. To counter this, checkpoint inhibitors, mainly monoclonal antibodies like ipilimumab, atezolizumab, and cemiplimab, have been developed. However, these drugs can have serious side effects, including myocarditis, diabetes, and hepatitis [6,7].

Myocarditis is a rare disorder characterized by inflammation of the myocardium, potentially reducing the heart's ability to efficiently pump blood. This condition, known as immune myocarditis, is more common than previously thought among cancer patients treated with ICIs [8].

According to statistics obtained between 2015 and 2017 from the National Cancer Institute, it was estimated that approximately 39.5% of men and women will be diagnosed with cancer at some point in their lives, a figure that may be higher today [9]. Consequently, a similar number of people are exposed to cancer treatments and to the potential adverse effects of these treatments. This article will evaluate how checkpoint inhibitors combat cancer cells while also potentially causing severe adverse effects, such as myocarditis.

## Review

Checkpoints

Understanding immune checkpoints will clarify why their inhibition is necessary and how it relates to treating diseases like cancer. Immune checkpoints are essential proteins in the immune system that help regulate the intensity and duration of immune responses. The most common places to find these checkpoints are on T cells, created in the bone marrow, and other tumor cells [10]. There are a variety of checkpoints; however, the main therapeutic targets that drugs bind to are CTLA-4, PD-1, and PD-L1. More recently, other targets have been investigated that are emerging as potential future avenues for the treatment of various tumors, such as VISTA, TIGIT, LAG-3, and TIM-3 [11,12]. The functionality of checkpoints is valuable for the immune system. However, diseases like cancer can disrupt their original functions, allowing them to go unnoticed by the immune system; thus, the disease is not counteracted [13].

Checkpoint inhibitors

Monoclonal antibodies are lab-created proteins designed to recognize specific targets in the body, enabling them to combat a wide range of cancers and other immune system diseases such as lymphomas and myeloma. These drugs are typically administered intravenously or, in some cases, intramuscularly. They are engineered to have high affinity and specificity for a particular antigen, allowing them to bind to various checkpoints in the immune system [14].

The first ICI, ipilimumab, was approved by the FDA in 2011 for melanoma treatment and was found to be effective. This breakthrough paved the way for further research and the development of more monoclonal antibodies. Unlike earlier drugs aimed at boosting immune activity, checkpoint inhibitors also sought to overcome immunosuppression. The creation of monoclonal antibodies has increased annually due to their effectiveness, with scientists continually refining these treatments to reduce side effects and address the rising incidence of diseases like cancer [2,15].

**Figure 1:**
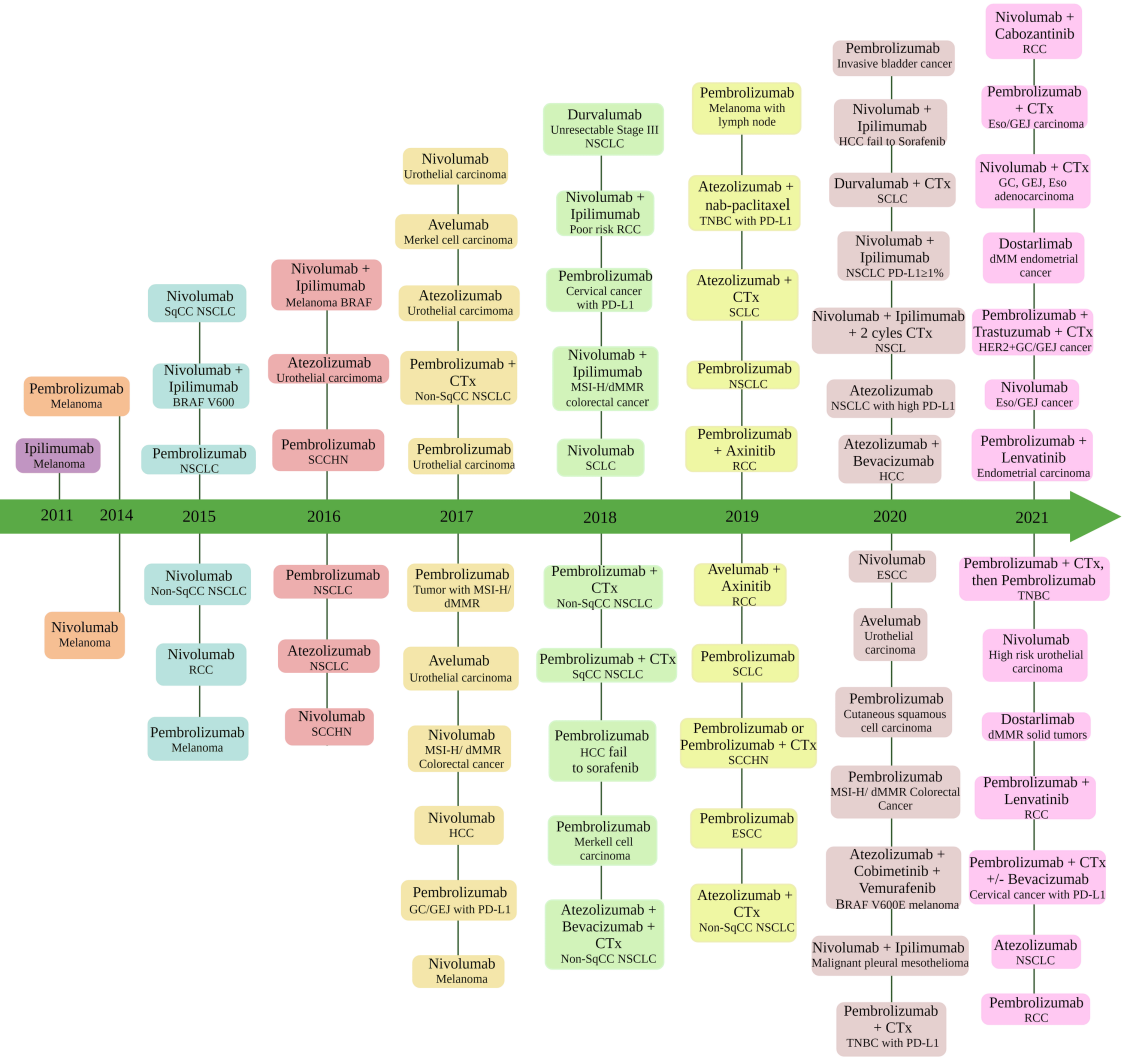
Medications approved by the FDA over the years NSCLC: non-small cell lung cancer; PD-L1: programmed cell death ligand 1; SqCC: squamous cell carcinoma; NonSqCC: non-squamous cell carcinoma; RCC: renal cell carcinoma; TNBC: triple negative breast cancer; HCC: hepatocellular carcinoma; Eso: esophageal; GC: gastric cancer; GEJ: gastroesophageal junction; ESCC: esophageal squamous cell carcinoma; CTx: chemotherapy; SCLC: small cell lung cancer; SCCHN: squamous cell carcinoma of the head and neck; MSI-H: microsatellite instability high; dMMR: mismatch repair deficient Image Credit: [16]; published with permission

Figure 1 illustrates the increasing innovation and production of checkpoint inhibitors over the years, with graphs detailing the medications approved from 2011 to 2021 [16].

These drugs were developed to combat diseases like cancer, which has been the leading cause of death worldwide. Data from 2020 revealed that nearly 10 million deaths were attributed to cancer [17]. When it comes to treating cancer, patients have several options: traditional methods like chemotherapy and radiotherapy, as well as newer approaches such as immunotherapy and targeted therapies [18].

Checkpoint inhibitor drugs are pivotal in this context. PD-1, a glycoprotein on T lymphocytes, regulates their activation by binding with ligands PD-L1 and PD-L2. High levels of PD-L1 produced by some cancer cells enable them to evade immune detection and attack [19].

In addition, CTLA-4 inhibitors are significant. CTLA-4 is a transmembrane protein that interacts with ligands B7-2 and CD28 to regulate immune responses. Monoclonal antibodies such as ipilimumab target CTLA-4 to inhibit its function [19]. Regulatory T cells (Tregs) are also important, as they can prevent T-lymphocyte activation, allowing cancer cells to continue multiplying and spreading without being effectively targeted by the immune system [20].

**Figure 2:**
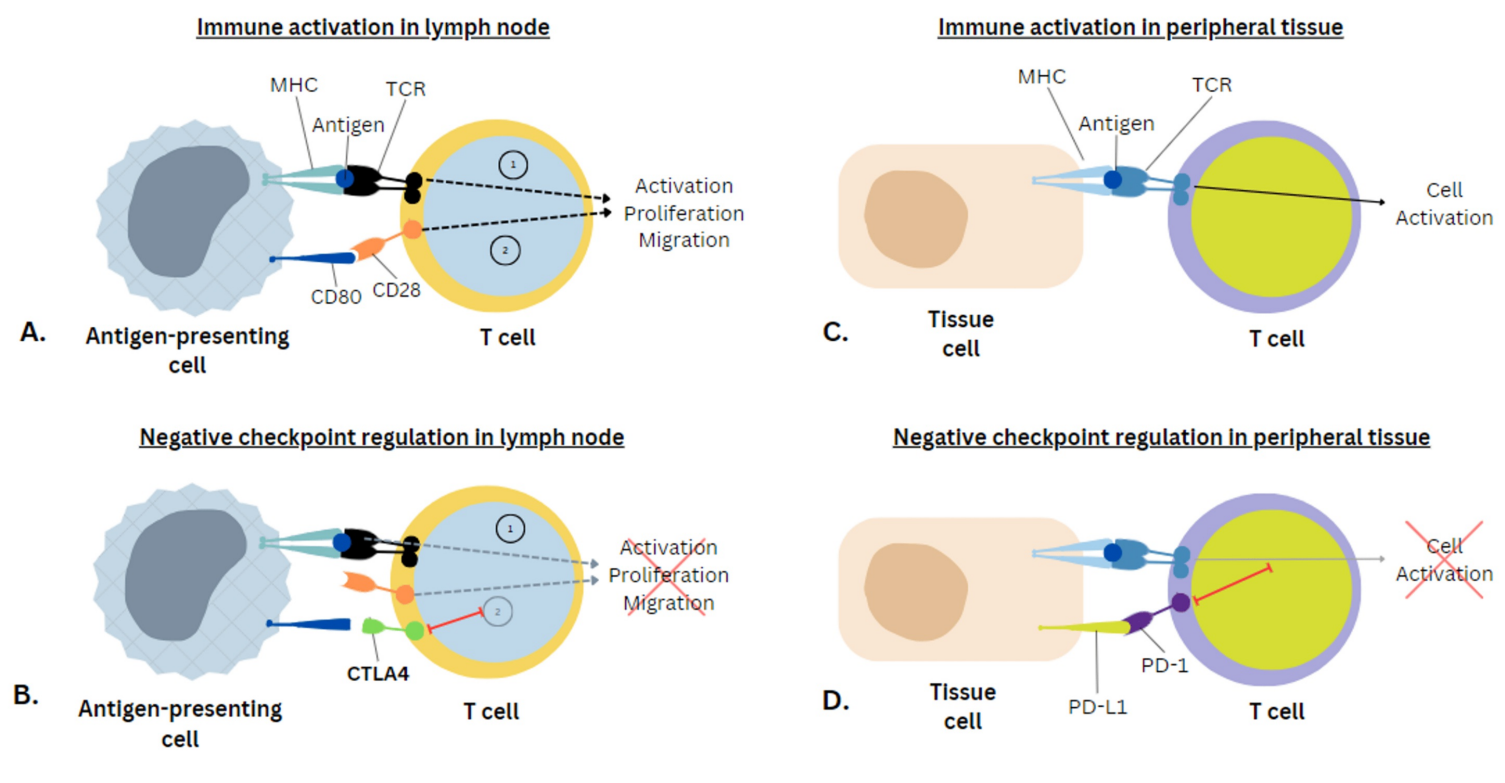
Immune activation and negative checkpoint regulation in lymph nodes and peripheral tissue (A) An antigen is presented on an antigen-presenting cell by way of an MHC molecule to a TCR on a T cell (Signal 1). In order to get immune activation, there has to be a co-stimulatory (Signal 2) that is shown when CD80 ligates with receptor CD28 which in turn causes the T cell to activate, proliferate, and migrate. (B) To avoid overwhelming activation of the immune system, checkpoints (CTLA-4) emerge to the surface of the cell to out-compete that co-stimulatory signal to get negative regulation and control that immune response. (C) Similar to A, T-cell activation starts when an antigen is presented to a TCR. (D) T cell expresses PD-1; additionally, the tissue has ligands (PD-L1) which are both checkpoints to decrease immune activation MHC: major histocompatibility complex; TCR: T-cell receptor; CTLA-4: cytotoxic T-lymphocyte antigen 4; PD-1: programmed cell death 1; PD-L1: programmed cell death ligand 1 Image Credit: [20]; published with permission

To correctly explain the mechanism of action of checkpoint inhibitors, it is important to demonstrate first how immune activation and negative checkpoint regulations work in the human body (Figure 2) [20].

**Figure 3:**
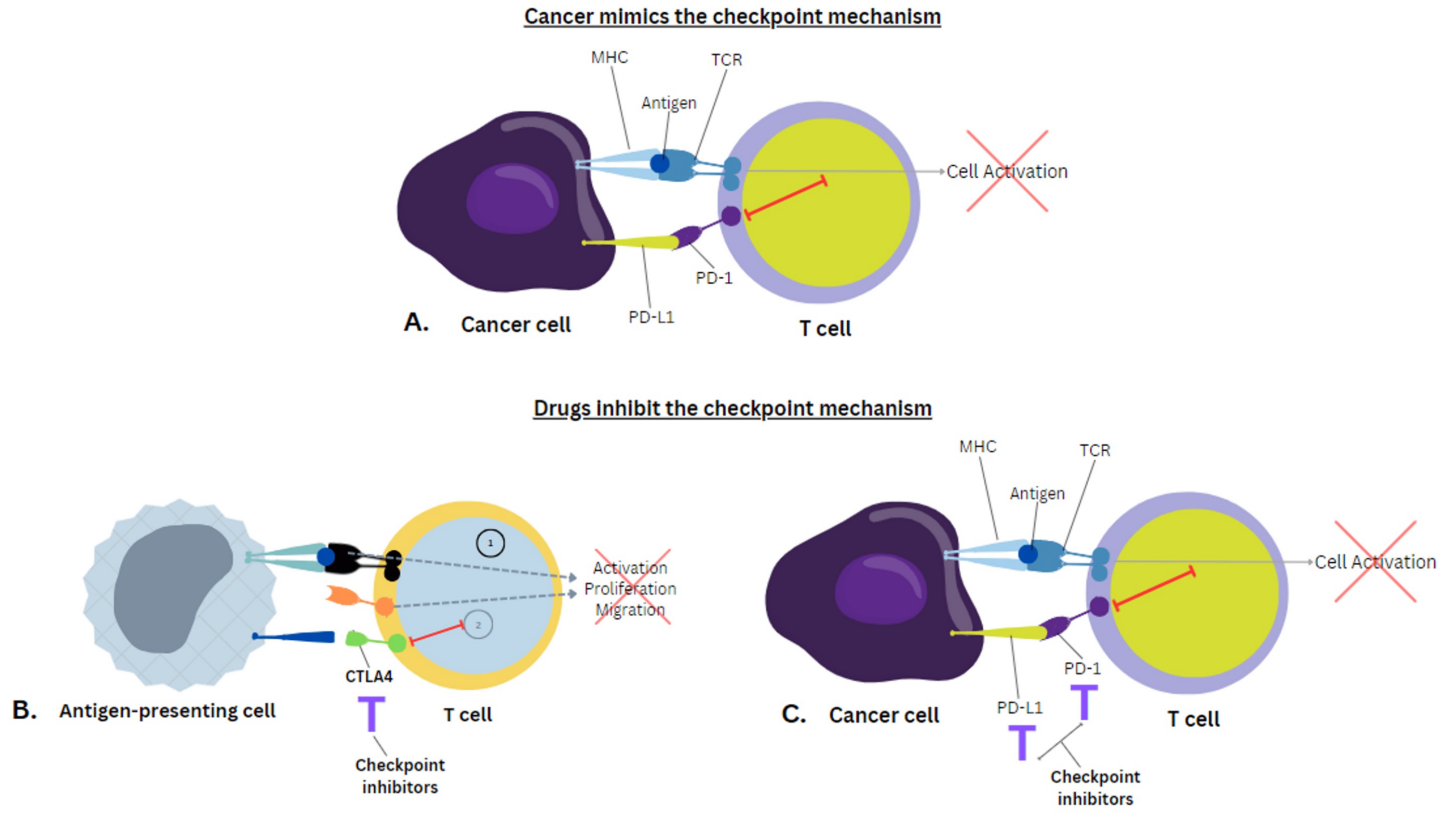
Mechanism of action of checkpoint inhibitors (A) Cancer cell finds a way to manipulate the immune system response, avoiding T-cell activation. (B) Drugs have been developed to inhibit CTLA-4 (checkpoint) as an adaptive immune system response, enabling activation of the T cell. (C) Drugs have been developed to inhibit PD-L1 and PD-1 (checkpoints) as an adaptive immune system response, enabling activation of the T cell MHC: major histocompatibility complex; TCR: T-cell receptor; CTLA-4: cytotoxic T-lymphocyte antigen 4; PD-1: programmed cell death 1; PD-L1: programmed cell death ligand 1 Image Credit: [20]; published with permission

Figure 3 explains the mechanism of action of checkpoint inhibitors in order to achieve an adaptive immune system activation [20]. 

Table 1 lists various ICIs along with their respective binding sites and target control points. Notable examples include recently FDA-approved PD-1 targeting agents such as tislelizumab, used as monotherapy for unresectable or metastatic esophageal squamous cell carcinoma (ESCC) [21], and toripalimab, indicated for nasopharyngeal carcinoma (NPC). Additionally, the FDA-approved drug relatlimab, which targets lymphocyte activation gene-3 (LAG-3), is used in combination with nivolumab (a PD-1 inhibitor) for melanoma treatment [22-24].

**Table 1:** Examples of checkpoint targets and some of their respective inhibitors Table Credit: [24]; published with permission

Inhibitors	Checkpoint target	Cardiotoxicity
Avelumab	PD-L1	Myocarditis
Atezolizumab	PD-L1	Myocarditis and vascular issues
Cemiplimab	PD-1	N/A
Durvalumab	PD-L1	Myocarditis
Ipilimumab	CTLA-4	Myocarditis, arrhythmias, and vascular issues
Pembrolizumab	PD-1	Myocarditis, vasculitis, and pericarditis
Dupilumab	IL-4Rα	Myocarditis
Nivolumab and relatlimab	PD-1/LAG-3	Myocarditis
Tislelizumab	PD-1	Myocarditis and pericarditis
Toripalimab	PD-1	Myocarditis, pericardial effusion, pericarditis, and vasculitis
Dostarlimab	PD-1	Myocarditis, pericarditis, and vasculitis
Tisagenlecleucel T	PD-1	N/A
Tremelimumab	CTLA-4	Cardiac arrhythmia, myocarditis, pericarditis, and vasculitis

Each monoclonal antibody targets different immune checkpoints, but they all share a common goal: to halt and eliminate cancer within the body. While these inhibitors have proven effective in treating various cancers, they also pose a potential risk of myocarditis, necessitating close clinical monitoring during their use.

Other checkpoint receptors, such as VISTA, TIGIT, and B7/H3, are being explored as potential drug targets. These receptors may offer new signaling pathways for regulating immune checkpoints and could become significant immunotherapies with potential clinical impact in the fight against various cancers [11,12].

Myocarditis

Like any pharmacological agent, checkpoint inhibitors possess the potential for adverse effects. These can manifest throughout the body, with the most prevalent being dermatologic toxicities, observed in 46-62% of patients, and autoimmune colitis. Table 2 lists various side effects caused by ICIs [23].

**Table 2:** Side effects of checkpoint inhibitors Table Credit: [23]; published with permission

Body system	Side effect
Gastrointestinal system	Hepatitis and colitis
Renal system	Nephritis
Cardiovascular system	Myocarditis, arrhythmias, and venous thromboembolism
Musculoskeletal system	Myositis and inflammatory arthritis

Conversely, there are rarer but more severe adverse effects, such as myocarditis, which can be life-threatening [24]. Myocarditis is an inflammation of the myocardium, the muscular layer of the heart. It can cause chest pain, arrhythmias, and severe left ventricular dysfunction. The most common causes of myocarditis are viral or bacterial infections. It can also be caused by cardiotoxic drugs or as an adverse effect of medications such as checkpoint inhibitors, B-lactams, or antiepileptics [25].

The incidence of myocarditis has been increasing in recent years due to various causes. A 2021 study estimated that approximately 1.8 million cases of myocarditis occur worldwide each year. These figures encompass myocarditis from all potential causes, highlighting its prevalence and rising incidence. Regardless of its etiology, myocarditis is a serious health concern and can be a leading cause of sudden death in young adults [26].

Table 3 presents a comparative chart showing the incidence of myocarditis related to checkpoint inhibitors, based on various studies conducted between 2016 and 2021 [27-31].

**Table 3:** Incidence of myocarditis associated with checkpoint inhibitors Original table

Authors	Year	One or two agents	Incidence of myocarditis
Johnson et al. [27]	2016	2	0.27%
Hu et al. [28]	2017	1	5.2%
Mahmood et al. [29]	2018	1	1.14%
Oren et al. [30]	2020	1	0.36%
Waliany et al. [31]	2021	1	1.4%

Myocarditis caused by checkpoint inhibitors can be difficult to detect, but certain altered parameters can indicate its presence. Elevated troponin levels are the primary biomarker, but changes in electrocardiograms and arrhythmias can also be detected [32]. A study by the Spanish Society of Cardiology included 105 patients, 35 of whom developed myocarditis due to the medication. This issue arose within three months and was identified by a 94% increase in troponin levels and electrocardiographic alterations in 89% of cases. The study concluded that myocarditis was more common in patients with cardiovascular risk, with a prevalence of 1.14% observed in this cohort [33].

Table 4 details the main biomarkers associated with myocarditis [34].

**Table 4:** Tests and biomarkers associated with myocarditis CKMB: creatine kinase-MB Table Credit: [34]; published with permission

Biomarker/test	Result
Troponin	Elevated, indicating damage to the heart muscle
C-reactive protein	Elevated, indicating inflammation
CKMB	Elevated, indicating damage to the heart muscle
Electrocardiogram	Irregular heartbeats (dysrhythmias)
Echocardiogram	Abnormalities in segmental wall motion
Myocardial biopsy	Inflammatory and infiltrative cardiomyopathies

A study at the Stanford Cancer Institute followed 214 patients undergoing checkpoint inhibitor therapy for nine months, with laboratory tests every 2-4 weeks to monitor troponin levels. Among these patients, 24 had abnormal troponin results, with three cases attributed to myocarditis induced by checkpoint inhibitors. Despite this issue, all three cases shared a positive outcome: the myocarditis was reversed within 4-8 months with high-dose glucocorticoid treatment [31]. However, myocarditis can lead to severe complications such as shock, cardiac arrest, ventricular tachycardia, and sudden death [29].

Treatment for myocarditis

Glucocorticoids have proven effective in treating myocarditis caused by checkpoint inhibitors, although much higher doses are required compared to other inflammatory conditions. Typically, chronic inflammatory adverse effects in organs are treated with 0.5-2.0 mg/kg of glucocorticoids such as prednisone. However, for myocarditis, up to 1 g daily of methylprednisolone may be used [25]. In some cases, both oral prednisone and intravenous methylprednisolone are prescribed simultaneously, with physicians administering up to 1000 mg orally daily along with 1 mg/kg/day of intravenous medication [35].

While glucocorticoids have been the primary treatment for myocarditis caused by checkpoint inhibitors, other alternatives exist. Medications for heart failure, such as beta-blockers, spironolactone, diuretics, and angiotensin-converting enzyme inhibitors, are options. Although antivirals have been tried, there is no conclusive data supporting their efficacy [36]. In severe cases where myocarditis leads to heart failure or arrhythmias, treatments like pacemakers, left ventricular assist devices, or even heart transplants may be considered [37]. In non-steroidal treatments, abatacept (a CTLA-4 agonist) shows the most promising data, with two randomized controlled trials currently evaluating its effectiveness in treating ICI myocarditis [38]. Additionally, rituximab, a monoclonal antibody, does not trigger myocarditis or other immune side effects and is used to treat complications caused by checkpoint inhibitors [39].

Some patients experience refractory cases of myocarditis caused by checkpoint inhibitors. The first line of treatment is glucocorticoids, particularly methylprednisolone. Studies conducted by Massachusetts General Hospital have shown that using high doses of these drugs can reduce the risk of major cardiovascular events by 73% compared to lower doses. In cases where the condition remains refractory and there is no improvement, other immunosuppressive agents such as mycophenolate mofetil or anti-thymocyte globulin (ATG) may be considered [40].

**Figure 4:**
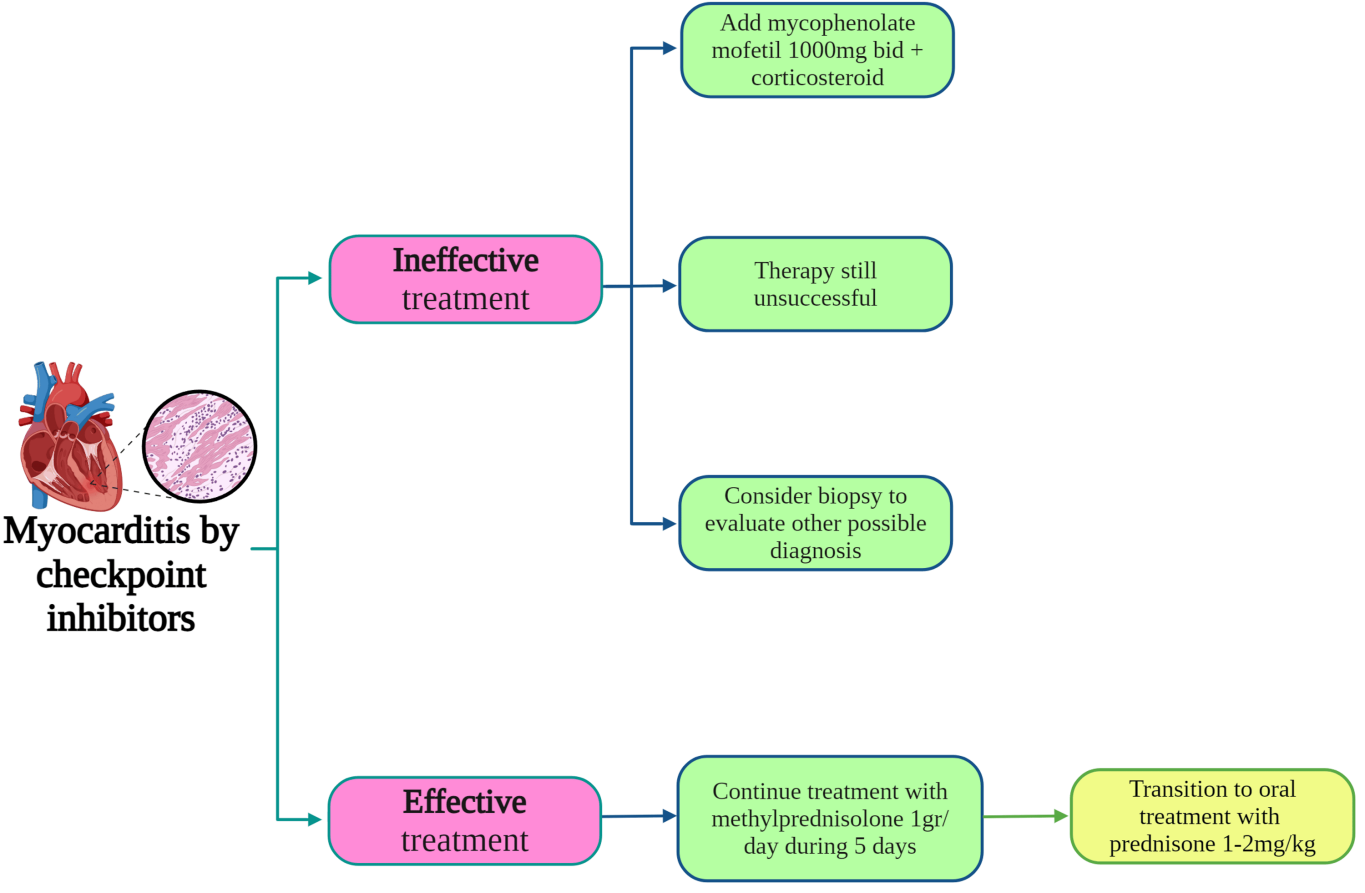
Treatment procedure for patients with refractory cases of myocarditis BID: twice daily Image Credit: [40]; published with permission

Figure 4 is a diagram showing the different treatment pathways that can be used depending on the patient's condition once they develop myocarditis [40].

Future research

Besides corticosteroids, several other immunosuppressive treatments, mainly targeting T lymphocytes, have been evaluated in patients with myocarditis induced by ICIs. These treatments include abatacept and Janus kinase (JAK) inhibitors like tofacitinib and ruxolitinib. Abatacept, a fusion protein that binds to CD80/CD86 on antigen-presenting cells and induces T-cell anergy, has demonstrated potential in preclinical studies. Nonetheless, its slow onset might not be suitable for rapidly progressing, life-threatening myocarditis cases. Current research focuses on optimizing abatacept's use, including a phase II trial investigating different dosages to achieve sufficient CD86 receptor occupancy. Moreover, combining abatacept with faster-acting immunosuppressors such as ruxolitinib is being explored to improve treatment outcomes. More studies are needed to identify the most effective treatment strategies and monitoring approaches for patients with ICI-induced myocarditis [41].

## Conclusions

Checkpoint inhibitors have garnered significant attention for their ability to treat various cancers by boosting the immune system's response against tumor cells. Despite their therapeutic successes, these drugs are associated with serious adverse effects, one of the most concerning being myocarditis. This condition, although previously underrecognized, has become increasingly common with the use of checkpoint inhibitors. Its incidence continues to rise annually, posing a substantial challenge for both prevention and management.

The precise mechanisms behind checkpoint inhibitor-induced myocarditis remain unclear, complicating efforts to prevent this severe complication. The increasing frequency of this adverse effect underscores the difficulty in developing effective preventive strategies. As our understanding of the underlying causes of myocarditis evolves, it remains a critical area for ongoing research. This research is essential to identify at-risk patients and to develop targeted approaches for mitigating the risk of myocarditis associated with checkpoint inhibitors.

Among the various treatment options for checkpoint inhibitor-induced myocarditis, high-dose corticosteroids have emerged as the most effective remedy. These steroids have shown promising results in reversing inflammation and improving heart function in affected patients. By suppressing the excessive immune response triggered by the checkpoint inhibitors, high-dose corticosteroids help alleviate the severe symptoms of myocarditis. However, their use must be carefully managed due to potential side effects. Continued research is needed to refine treatment protocols and to improve the overall understanding of this condition, ensuring better outcomes for patients undergoing checkpoint inhibitor therapy.

## References

[1] Nicholson LB (2016). The immune system. Essays Biochem.

[2] Ibis B, Aliazis K, Cao C, Yenyuwadee S, Boussiotis VA (2023). Immune-related adverse effects of checkpoint immunotherapy and implications for the treatment of patients with cancer and autoimmune diseases. Front Immunol.

[3] (2024). Immune control point inhibitors. https://www.cancer.gov/espanol/cancer/tratamiento/tipos/inmunoterapia/inhibidores-de-puntos-de-control.

[4] Jenkins RW, Barbie DA, Flaherty KT (2018). Mechanisms of resistance to immune checkpoint inhibitors. Br J Cancer.

[5] (2024). Immune checkpoint inhibitors and side effects. https://www.cancer.org/es/cancer/como-sobrellevar-el-cancer/tipos-de-tratamiento/inmunoterapia/inhibidores-puestos-de-control-inmunitarios.html.

[6] Lee S, Dy G (2020). Estado actual de la inmunoterapia en las neoplasias malignas torácicas. Gea consultoría editorial s.l. Inhibidores del punto de control inmunitario en oncología.

[7] Hu JR, Florido R, Lipson EJ (2019). Cardiovascular toxicities associated with immune checkpoint inhibitors. Cardiovasc Res.

[8] Patel RP, Parikh R, Gunturu KS, Tariq RZ, Dani SS, Ganatra S, Nohria A (2021). Cardiotoxicity of immune checkpoint inhibitors. Curr Oncol Rep.

[9] (2024). Cancer statistics. https://www.cancer.gov/espanol/cancer/naturaleza/estadisticas.

[10] García-Ramos SE, García-Poza P, Ramos-Díaz F (2024). Update in monoclonal antibody therapy. Published Online First.

[11] Cai L, Li Y, Tan J, Xu L, Li Y (2023). Targeting LAG-3, TIM-3, and TIGIT for cancer immunotherapy. J Hematol Oncol.

[12] Wu C, Cao X, Zhang X (2021). VISTA inhibitors in cancer immunotherapy: a short perspective on recent progresses. RSC Med Chem.

[13] (2024). Inhibidores de puntos de control inmunitario en la terapia del cáncer. https://idus.us.es/handle/11441/132557.

[14] (2024). Monoclonal antibodies. https://www.cancer.gov/espanol/cancer/tratamiento/tipos/inmunoterapia/anticuerpos-monoclonales.

[15] Alexander W (2016). The checkpoint immunotherapy revolution: what started as a trickle has become a flood, despite some daunting adverse effects; new drugs, indications, and combinations continue to emerge. P T.

[16] Lee JB, Kim HR, Ha SJ (2022). Immune checkpoint inhibitors in 10 years: contribution of basic research and clinical application in cancer immunotherapy. Immune Netw.

[17] (2024). Cancer. https://www.who.int/es/news-room/fact-sheets/detail/cancer.

[18] Shahzad HN (2018). Neoplasm. IntechOpen.

[19] Cárdenas-Oyarzo AM, Bocchieri-Oyarce PA, Méndez-Laport CR, Zolezzi JM, Ríos JA (2022). Immune checkpoint inhibitors. A breakthrough in cancer therapy [Article in Spanish]. Rev Med Chil.

[20] Bermejo Toscano Á (2024). Inhibidores de puntos de control inmunitario en la terapia del cáncer. https://idus.us.es/bitstream/handle/11441/132557/BERMEJO%20TOSCANO%20ALVARO.pdf?sequence=1.

[21] (2024). FDA approves tislelizumab for unresectable or metastatic ESCC. https://www.targetedonc.com/view/fda-approves-tislelizumab-for-unresectable-or-metastatic-escc.

[22] (2024). FDA approves toripalimab-tpzi for nasopharyngeal carcinoma. FDA. Published Online First: 30 October.

[23] Westfield C (2024). Managing immune checkpoint inhibitor side effects: key recommendations. https://oncpracticemanagement.com/issues/2018/may-2018-vol-8-no-5/678-managing-immune-checkpoint-inhibitor-side-effects-key-recommendations.

[24] Heinzerling L, de Toni EN, Schett G, Hundorfean G, Zimmer L (2019). Checkpoint inhibitors. Dtsch Arztebl Int.

[25] Moslehi J, Lichtman AH, Sharpe AH, Galluzzi L, Kitsis RN (2021). Immune checkpoint inhibitor-associated myocarditis: manifestations and mechanisms. J Clin Invest.

[26] Golpour A, Patriki D, Hanson PJ, McManus B, Heidecker B (2021). Epidemiological impact of myocarditis. J Clin Med.

[27] Johnson DB, Balko JM, Compton ML (2016). Fulminant myocarditis with combination immune checkpoint blockade. N Engl J Med.

[28] Hu YB, Zhang Q, Li HJ (2017). Evaluation of rare but severe immune related adverse effects in PD-1 and PD-L1 inhibitors in non-small cell lung cancer: a meta-analysis. Transl Lung Cancer Res.

[29] Mahmood SS, Fradley MG, Cohen JV (2018). Myocarditis in patients treated with immune checkpoint inhibitors. J Am Coll Cardiol.

[30] Oren O, Yang EH, Molina JR, Bailey KR, Blumenthal RS, Kopecky SL (2020). Cardiovascular health and outcomes in cancer patients receiving immune checkpoint inhibitors. Am J Cardiol.

[31] Waliany S, Neal JW, Reddy S (2021). Myocarditis surveillance with high-sensitivity troponin I during cancer treatment with immune checkpoint inhibitors. JACC CardioOncol.

[32] Abdolall AK, Srivastava G, Abdelnour J, Balakumaran K, Aneja A (2023). Immune checkpoint inhibitor associated myocarditis. J Am Coll Cardiol.

[33] Revilla M (2024). Myocarditis in patients treated with immune checkpoint inhibitors. Soc. Esp. Cardiol..

[34] Dominguez F, Kühl U, Pieske B, Garcia-Pavia P, Tschöpe C (2016). Update on myocarditis and inflammatory cardiomyopathy: reemergence of endomyocardial biopsy. Rev Esp Cardiol (Engl Ed).

[35] Palaskas N, Lopez-Mattei J, Durand JB, Iliescu C, Deswal A (2020). Immune checkpoint inhibitor myocarditis: pathophysiological characteristics, diagnosis, and treatment. J Am Heart Assoc.

[36] Guglin M, Nallamshetty L (2012). Myocarditis: diagnosis and treatment. Curr Treat Options Cardiovasc Med.

[37] (2024). Myocarditis. https://www.nhlbi.nih.gov/health/heart-inflammation/myocarditis.

[38] (2024). AbataCept for the Treatment of Immune-cHeckpoint Inhibitors Induced mYocarditiS (ACHLYS). https://clinicaltrials.gov/study/NCT05195645.

[39] (2024). UpToDate Lexidrug content sets and tools. https://www.wolterskluwer.com/en/solutions/uptodate/enterprise/lexidrug-content-sets-and-tools.

[40] Frayberg M, Yung A, Zubiri L, Zlotoff DA, Reynolds KL (2021). What the cardiologist needs to know about cancer immunotherapies and complications. Curr Treat Options Oncol.

[41] Moslehi J, Salem JE (2022). Immune checkpoint inhibitor myocarditis treatment strategies and future directions. JACC CardioOncol.

